# The species of the Neotropical genus *Fractipons* Townes, 1970 (Hymenoptera,  Ichneumonidae, Cryptinae)

**DOI:** 10.3897/zookeys.76.913

**Published:** 2011-01-19

**Authors:** Santiago Bordera, Alejandra González-Moreno

**Affiliations:** Instituto de Investigación CIBIO (Centro Iberoamericano de Biodiversidad), Universidad de Alicante, Apdo. Corr. 99, 03080, Alicante, Spain

**Keywords:** *Fractipons*, new species, Hymenoptera, Ichneumonidae, Cryptinae, taxonomy, key

## Abstract

In this paper, two new species of the Neotropical genus Fractipons Townes, 1970 (Hymenoptera, Ichneumonidae) are described. A new diagnosis for the genus, a re-description of Fractipons cincticornis Townes, 1970 and a key to known species are provided. New distribution records for the genus now include Argentina, Costa Rica, Panama and Peru.

## Introduction

**Figures 1–3.**
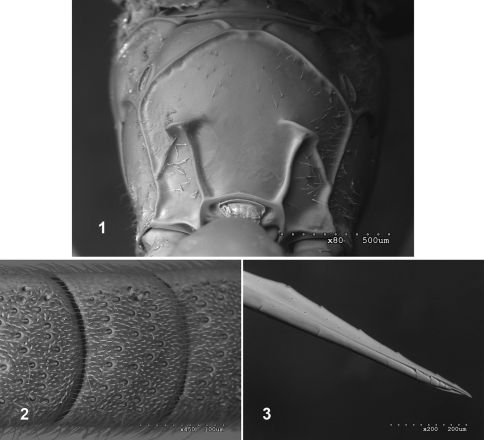
Fractipons spp. **1** Propodeum of Fractipons dasyscutum sp. n., dorsal view **2** Ventral flat part of female flagellum of Fractipons cincticornis **3** Ovipositor tip of Fractipons cincticornis, lateral view.

Fractipons Townes, 1970 is a small, little known Neotropical genus of Ichneumonidae of the subfamily Cryptinae belonging to the Chiroticina sensu Townes (1970). This subtribe was considered by Townes to be the most ‘natural’ of the subtribes, but subtribes of Phygadeuontini are currently not recognised (Yu et al. 2005) and recent studies based on molecular methods discourage the use of the traditional subtribes of Townes (Laurenne et al. 2006). Nevertheless, no new regrouping of genera based on phylogenetic criteria has been proposed for Phygadeuontini, so we consider “Chiroticina” sensu Townes as a framework for the taxonomic position of Fractipons, based on the presence of an isolated mesopleural pit, which is the strongest single feature that characterizes this group (Townes 1970). Fractipons is close to Epelaspis Townes, 1970 and Mamelia Seyrig, 1952, in having the genal portion of the occipital carina reaching the base of the mandible, the median lobe of the mesoscutum without a median longitudinal groove, and the posterior transverse carina of the mesosternum interrupted in front of the mid coxae, but Fractipons is easily distinguishable from them by the apical transverse carina of the propodeum, strong and abruptly interrupted in the centre and forming lateral flat crests (Fig. 1). There is only one described species, the type species Fractipons cincticornis Townes, 1970from Brazil (Townes 1970), but Townes also mentioned two more species that remained undescribed. Nothing is known about the biology of this genus. The aim of this work is to describe these two new species and to provide a key to the known species.

## Material and methods

In this work, sixty-five specimens preserved in the American Entomological Institute (Gainesville, Florida, USA), in the Florida State Collection of Arthropods (Department of Agriculture, Gainesville, Florida, USA) and in INBio (Santo Domingo de Heredia, Costa Rica), including type material of Fractipons cincticornis, have been studied. Morphological terminology follows Gauld (1991). Measurements used in descriptions were made as follows: head width is the maximum distance between the outline of the eyes in dorsal view; head length is measured from the anterior edge of the eye to the hind edge of the gena; body length is approximate because specimens are rarely in a natural position on pins (in females, ovipositor length is excluded). For the same reason we did not measure the length of the metasoma. Terminology used for describing body surface sculpture is based on Harris (1979). Townes (1970) described Fractipons based on his Lissaspis description, pointing out the differing characters. We provide a complete characterization of the genus based on Townes (1970) and new features.

Images were made with an Olympus M1060 digital camera attached to a Leica MZ12 stereomicroscope. The SEM images were taken using an Hitachi S-3000N (in low vacuum mode) in the University of Alicante, Spain.

The master map for distribution area was downloaded from http://picses.eu/image/8730bd0d/

Type material is deposited in the entomological collections of the American Entomological Institute (AEIC), the Instituto Nacional de Biodiversidad (INBio), University of Alicante (Alicante, Spain, CEUA) and in the Florida State Collection of Arthropods (FSCA).

## Results

### Fractipons

Townes, 1970

#### Type species:

Fractipons cincticornis Townes, 1970. Memoirs of the American Entomological Institute 12: 14. Holotype, ♀.

#### Diagnosis.

Mesopleural impression below speculum consisting of an isolated pit which is some distance in front of mesopleural suture. Occipital carina reaching base of mandible. Median lobe of mesoscutum without median longitudinal groove. Posterior transverse carina of the mesosternum interrupted in front of mid coxae. Apical transverse carina of propodeum strong, abruptly interrupted medially and forming lateral flat crests (Fig. 1).

#### Description.

Body moderately slender, 4.8–7.1 mm, mostly smooth and polished. Head transverse. Flagellomeres of female conspicuously thickened from third flagellomere, slightly thin towards apex, from tenth to penultimate flattened below; in this flat area with conspicuous setiferous sensillae (Fig. 2). Lower face finely and densely punctate, with small central prominence. Clypeus rather wide, apical margin sharp, straight or slightly arcuate. Malar space forming wide and deep granulate area (Figs 16, 18, 20). Mandible moderately tapered to apex, lower tooth shorter than upper tooth. Maxillary palpus reaching to ventral part of epicnemial carina. Occipital carina joining base of mandible, nearly angular on mid-dorsal part. Pronotal transverse groove without median longitudinal ridge. Epomia absent. Median lobe of mesoscutum without median longitudinal groove. Notauli rather weak, about 0.3–0.7 as long as mesoscutum. Precutellar groove without traces of longitudinal carinae. Scutellum moderately convex, polished and smooth or very sparsely punctate, lateral carinae strong, extending about 0.8–0.9 its length. Mesopleuron completely smooth and polished.Mesopleural impression below speculum consisting of an isolated pit some distance in front of mesopleural suture. Sternaulus weak on anterior 0.3–0.5, nearly absent posteriorly. Epicnemial carina reaching 0.7–0.9 × height of mesopleuron, at upper margin weak or absent. Posterior transverse carina of mesosternum widely interrupted in front of each mid coxa, laterally elevated forming strong flat crest. Areolet open. Ramulus absent. Vein 2*m–cu* weakly inclivous, with two bullae. Vein *cu-a* opposite *Rs+M* or slightly basal. Hind wing with *M+Cu* moderately curved at apical 0.5. Abscissa of *M+Cu* between *M* and *Cu_1_* longer than *cu-a*, strongly inclivous*, cu-a* reclivous. Propodeum with anterior transverse carina strong and complete. Apical transverse carina of propodeum strong and abruptly interrupted medially, forming lateral flat crests (Fig. 1). Lateral longitudinal carina of propodeum only present apically, distad of crests. Lateromedian carina partially present in area basalis. Area superomedia absent. Pleural carina rounded and strong. Submetapleural carina forming anterior flat crest. Juxtacoxal carina absent. Propodeal spiracle elongate. First metasomal tergite smooth and polished, sometimes with sparse setiferous punctures, dorsally, laterally, upper face weakly convex, median dorsal and lateral carinae absent. Spiracle at the apical 0.46. Postpetiole about 0.7–0.8 times as long as maximum width (measured dorsally). Tergites 2–7 smooth and shiny with fine setiferous punctures. Epipleura of tergites 2 and 3 separated by crease, of tergite 4 not separated. Gastrocoelus wider than long, thyridium finely granulate. Ovipositor straight, with nodus, upper valve with five dorsal teeth, lower valve with three oblique notches and 4–5 small complete and transverse apical teeth (Fig. 3).

#### Key to the species of Fractipons.

**Table:** 

1	Females (with conspicuous ovipositor)	2
–	Males	4
2	Mesoscutum with very dense setae (Fig. 10). Malar space about 0.7–0.8 times width of mandible base (Fig. 16). Lower rim of mandible expanded at base with rounded translucent area (Fig. 16)	Fractipons dasyscutum sp. n.
–	Mesoscutum smooth and shiny, sometimes with sparse, short setae (Fig. 12) or more dense, long setae (Fig. 14). Malar space about 0.5 times width of mandible base (Figs 18, 20). Basal, lower rim of mandible without conspicuous translucent area	3
3	Head black, mesosoma and metasoma orange (Fig. 4). Occipital carina conspicuously elevated, at least in genal section (Fig. 18). Mesoscutum with moderately sparse, long setae (Fig. 14)	Fractipons cincticornis Townes
–	Body entirely yellow-orange (Fig. 7). Occipital carina not conspicuously elevated (Fig. 20). Mesoscutum smooth, sometimes with short and sparse setae (Fig. 12)	Fractipons glabriusculus sp. n.
4	Flagellum black with white band on segments 7(8)–12(13). Head black, mesosoma and metasoma orange, sometimes front part of mesosoma brownish (Fig. 5)	Fractipons cincticornis Townes
–	Flagellum black, dark brown or partially orange at base, never with a white band. Body entirely orange (Figs 6, 9)	5
5	Mesoscutum with very dense setae (Fig. 11). Head in lateral view with upper part of gena straight and abruptly reduced (Fig. 24). Malar space about 0.6–0.8 times width of mandible base (Fig. 17). Lower rim of mandible expanded at base, with rounded, translucent area (Fig. 17).	Fractipons dasyscutum sp. n.
–	Mesoscutum smooth and shiny, sometimes with some short, sparse setae anteriorly (Fig. 13). Head in lateral view with upper part of gena rounded. (Fig. 25). Malar space about 0.3–0.4 times width of mandible base (Fig. 21). Basal, lower rim of mandible without conspicuous translucent area	Fractipons glabriusculus sp. n.

### Fractipons cincticornis

Townes, 1970

Fractipons cincticornis  Townes, 1970. Memoirs of the American Entomological Institute 12: 14. Holotype, ♀.

#### Diagnosis.

**Figures 4–9.**
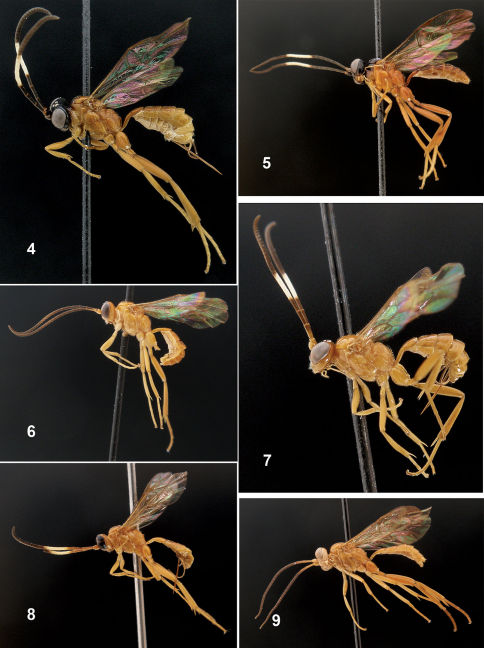
Habitus of Fractipons spp., lateral view. **4–5** Fractipons cincticornis **4** female **5** male. **6–7** Fractipons glabriusculus sp. n. **6** paratype male **7** holotype female. **8–9** Fractipons dasyscutum sp. n. **8** holotype female **9** paratype male.

Mesoscutum smooth and shiny, with moderately sparse long setae (Figs 14, 15).Malar space about 0.4–0.5 times width of mandible base (Figs 18, 20). Basal lower rim of mandible without conspicuous translucent area. Head black, mesosoma and metasoma orange, sometimes front part of mesosoma brownish. Both male and female with white band on flagellomeres 7(8)–12 (13) and 4–8, respectively (Figs 4, 5). Occipital carina conspicuously elevated, at least in ventral section (Figs 18, 19).

#### Description.

##### Female:

Body length 6.0–7.1 mm. Head 0.8–0.9 mm long, 1.4–1.8 mm wide. Mesosoma 2.2–2.8 mm long, 1.0–1.3 mm wide (mesoscutum). Fore wing 4.7–6.0 mm long. Petiole 1.1–1.5 mm long. Ovipositor sheath 1.5–2.1 mm long.

###### Head:

Transverse, 1.7–1.9 times as wide as long, mostly smooth and shiny, strongly constricted behind compound eyes. Antenna with 26–28 flagellomeres, conspicuously thickened from third flagellomere, slightly thin towards apex. First flagellomere 4.7–6.0 times as long as maximum width; flagellomeres from tenth to penultimate flattened below; in this flat area with conspicuous setiferous sensillae (Fig. 2). Gena 0.2–0.3 times as long as eye (in dorsal view), with fine and dense setiferous punctures on lower half, upper part in lateral view nearly straight, strongly constricted. Occiput strongly depressed in centre. Lower face finely and densely punctate, with small central prominence, clypeus rather wide, almost flat, apical margin straight or slightly arcuate. Malar space with wide granulate groove, about 0.5–0.6 times width of mandible base (Fig. 18). Posterior ocellus separated from eye by about 1.2–1.3 times its diameter. Space between posterior ocelli 0.6–0.8 times their diameter. Occipital carina reaching base of mandible, conspicuously elevated, at least in genal section (Fig. 18), nearly angular medially, dorsally. Mandible moderately tapered to apex, lower tooth shorter than upper tooth, finely granulate on basal half (Fig. 18). Maxillary palpus reaching to ventral part of epicnemial carina.

###### Mesosoma:

Pronotal transverse groove without median longitudinal ridge. Epomia absent. Mesoscutum smooth and shiny with moderately sparse long setae (Fig. 14). Median lobe of mesoscutum without median longitudinal groove. Notauli slightly indicated anteriorly. Prescutellar groove without traces of longitudinal carinae. Scutellum moderately convex, polished and smooth or very sparsely punctate, lateral carinae strong, extending about 0.8–0.9 its length. Mesopleuron completely smooth and polished. Mesopleural impression below speculum consisting of an isolated pit some distance in front of mesopleural suture. Sternaulus weak on anterior 0.3–0.4, nearly absent posteriorly. Epicnemial carina reaching 0.8 times height of mesopleuron, weak or absent dorsally. Posterior transverse carina of mesosternum widely interrupted in front of each mid coxa, laterally elevated as flat low crest. Areolet of fore wing open. Marginal cell 2.8–3.0 times as long as deep. Ramulus absent. Vein 2*m–cu* weakly inclivous, with two bullae. Vein *cu-a* opposite *Rs+M* or slightly basal. Abscissa of *Cu_1_* between1*m-cu* and *Cu_1a_* 1.6–1.9 times length of *Cu_1b_*, both clearly inclivous. Hind wing with *M+Cu* moderately curved at apical 0.5. Abscissa of *M+Cu* between *M* and *Cu_1_* strongly inclivous, 1.1–1.3 times as long as *cu-a* which is strongly reclivous. Hind femur about 5.1–5.3 as long as high. Propodeum with anterior transverse carina strong and complete, posterior transverse carina centrally absent and forming broad, low, flat crest, lateral longitudinal carina only present apically, distad of crests. Lateromedian carina partially present in area basalis. Area superomedia absent. Pleural carina rounded and strong. Submetapleural carina forming an anterior strong, flat crest. Juxtacoxal carina absent. Propodeal spiracle strongly elongate.

###### Metasoma:

First metasomal tergite smooth and polished, sometimes with sparse setiferous punctures dorsally, laterally. Median dorsal and lateral carinae absent. Postpetiole about 0.7–0.8 times as long as maximum width (measured dorsally). Second and remaining tergites polished, with very weak dense setiferous punctures. Gastrocoelus wider than long, thyridium finely granulate. Ovipositor straight, with nodus and five dorsal apical teeth on upper valve, lower valve with three oblique notches and 4–5 small complete and transverse apical teeth (Fig. 3). Ovipositor sheaths 0.6–0.8 times as long as hind tibia.

###### Colour:

Mesosoma and metasoma entirely yellowish orange. Head dark brown to black (Fig. 4). Mandibles, except base and teeth, clypeus apically, scape and pedicel and usually two spots on frontal orbits yellow or orange. Flagellum brown to blackish with a white band on flagellomeres 4–8. Sometimes lower face partially orange tinged. Wing membrane with fine yellowish tinge (Fig. 4).

##### Male:

Body length 6.0–7.0 mm. Head 0.7–0.8 mm long and 1.3–1.6 mm wide. Mesosoma 2.0–2.6 mm long, 1.0–1.2 mm wide (maximum width of mesoscutum). Fore wing 4.8–5.3 mm long. Petiole 1.0–1.3 mm long.

Similar to female except as follows:

###### Head:

Transverse, 1.9–2.0 times as wide as long, moderately constricted behind compound eyes. Antenna with 27–28 segments. Flagellum filiform, slightly tapered towards apex, first flagellomere 5.1–5.7 times as long as maximum width. Tyloids narrow and elevated on flagellomeres 11(12)–13(14, 15), moderately wide at base (Figs 26, 27), with small secretory pores on top (see Isidoro et al. 1996; Bin et al. 1999; Bordera and Hernández-Rodríguez 2003; Steiner et al. 2010). Gena in dorsal view, rounded, 0.4–0.5 times as long as eye, upper part less constricted. Malar space about 0.4–0.5 times as wide as basal width of mandible (Fig. 19). Posterior ocellus separated from eye by about 1.3–1.5 times its diameter. Space between posterior ocelli 0.5–0.6 times their diameter.

###### Mesosoma:

Marginal cell 2.8–3.1 times as long as deep. Abscissa of *M+Cu* between *M* and *Cu_1_* strongly inclivous, 1.3–1.6 times as long as *cu-a*, which is strongly reclivous. Hind femur about 5.5–5.7 as long as high.

###### Metasoma:

Postpetiole 0.8–1.0 times as long as wide. Second and remaining tergites with dense, fine setiferous punctures.

###### Colour:

Antenna entirely dark brown with white ring on flagellomeres 7(8) –12(13). Head black, sometimes widely yellowish or orange on lower face and/or on scape and pedicel below and/or also with two orange spots on facial orbits and frontal orbits. Pronotum dorsally and meoscutum brown to dark brown. Metasoma sometimes from postpetiole to at least tergite 5 brown-orange. Wing membrane with fine yellowish tinge (Fig. 5).

**Figures 10–15.**
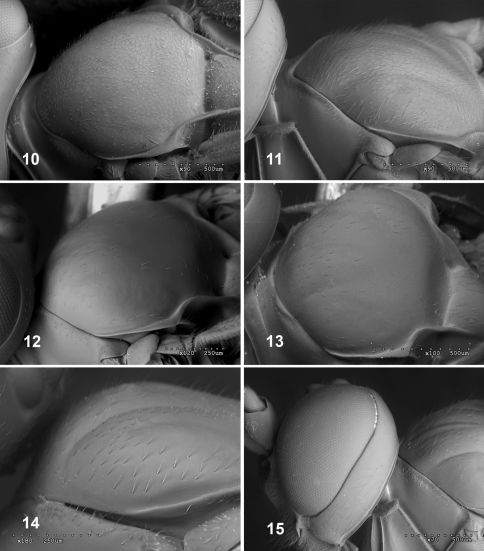
Mesosoma, dorsal view. **10–11** Fractipons dasyscutum sp. n. **10** female **11** male.**12–13** Fractipons glabriusculus sp. n.**12**female **13** male. **14–15** Fractipons cincticornis **14** female **15** male.

**Figures 16–21.**
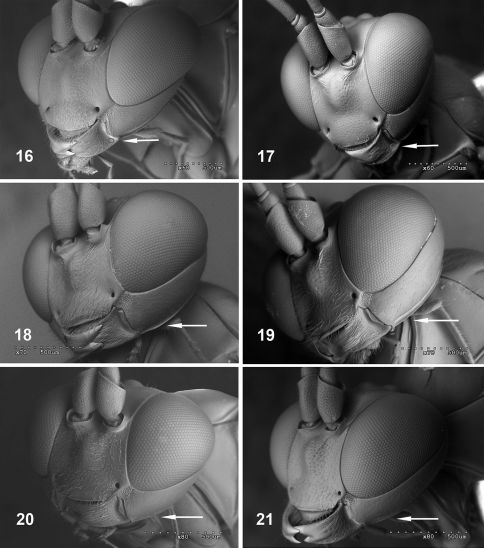
Head, frontal view. **16–17** Fractipons dasyscutum sp. n. (position of translucent area in mandible arrowed) **16** female **17** male. **18–19** Fractipons cincticornis (occipital carina arrowed)**18** female **19** male **20–21** Fractipons glabriusculus sp. n. (occipital carina arrowed)**20**female **21** male.

**Figures 22–25.**
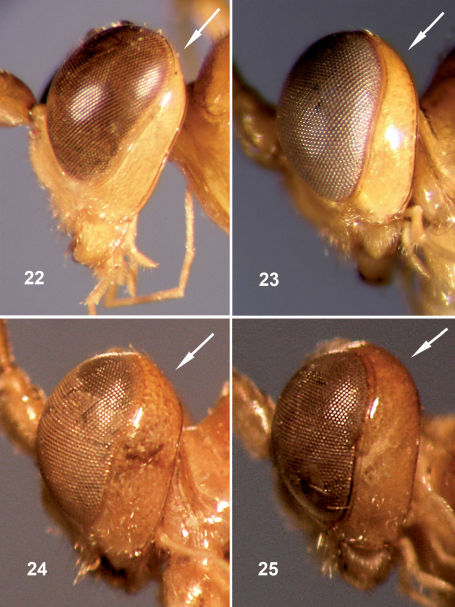
Head, lateral view (upper part of gena arrowed). **22–23** Females **22** Fractipons dasyscutum sp. n. **23** Fractipons glabriusculus sp. n.,**24–25** Males **24** Fractipons dasyscutum sp. n. **25** Fractipons glabriusculus sp. n.

#### Material examined.

**Type material.** Holotype female with labels as follows: Brazil, Teresópolis, 13-III-1966, H. & M. Townes (AEIC).Paratypes: Brazil, 1 ♂, Teresópolis, 9-III-1966, H. & M. Townes; 1 ♂, same locality, 10-III-1966, H. & M. Townes; 1 ♀, 2 ♂♂, same locality, 11-III-1966, H. & M. Townes; 1 ♂, Campiña Grande near Curitiba, 14-II-1966, H. & M. Townes; 1 ♀, 1 ♂, Rio de Janeiro, 5-III-1966, H. & M. Townes; 1 ♀, Nova Teutonia, Santa Catarina, 17-X-1952, Fritz Plaumann (all AEIC). **Non type material:** Brazil, 2 ♂♂, Represa Rio Grande, Guanabara, X-1969, M. Alvarenga; 2 ♂♂, same locality, IX-1969, M. Avarenga; 2 ♂♂, same locality, I-1972, M. Alvarenga (all AEIC); 1 ♂, S. Bocaina, 1650 m, S. J. Barreiro, XI-1968, Alvarenga & Seabra (CEUA).

#### Distribution.

Brazil (Fig. 32).

### Fractipons glabriusculus sp. n.

urn:lsid:zoobank.org:act:FF28BED0-FF1F-47B5-B9AB-F26DA1DD5953

#### Diagnosis.

Mesoscutum smooth and shiny, sometimes with some isolated, short setae (Figs 12, 13). Head in lateral view with upper part of gena rounded (Figs 23, 25). Female malar space 0.5 times width of mandible base (Fig. 20), in male 0.3–0.4 times (Fig. 21). Body entirely yellow–orange, rarely in males darkened dorsally (Figs 6, 7). Flagellum dark brown to black, in female with wide band on flagellomeres 4–7 (Fig. 7), male always without this band (Fig. 6). Occipital carina not conspicuously elevated (Figs 20, 21).

#### Description.

##### Female:

Body length 4.8–6.2 mm. Head 0.6–0.8 mm long, 1.2–1.5 mm wide. Mesosoma 1.8–2.1 mm long, 1.0–1.1 mm wide (mesoscutum). Fore wing 4.4–4.8 mm long. Petiole 1.0–1.1 mm long. Ovipositor sheath 1.4–1.5 mm long.

###### Head:

Transverse, 1.9 times as wide as long, mostly smooth and shiny, constricted and slightly rounded behind compound eyes. Antenna with 25 flagellomeres, conspicuously thickened from third flagellomere, slightly thin towards apex. First flagellomere 4.5–5.5 times as long as maximum width, flagellomeres from tenth to penultimate flattened below, in this flat area with conspicuous setiferous sensillae. Gena 0.25–0.35 times as long as eye (in dorsal view), upper part in lateral view rounded (Fig. 23). Occiput moderately depressed in centre. Lower face finely, densely punctate, with small central prominence, clypeus rather wide, weakly convex, apical margin slightly arcuate. Malar space with wide granulate groove, about 0.5 times width of mandible base (Fig 20). Posterior ocellus separated from eye by about 1.3–1.4 times its diameter. Space between posterior ocelli 0.6–0.7 times their diameter. Occipital carina joining base of mandible, not conspicuously elevated in genal region (Fig. 20), nearly angular medially, dorsally. Mandible moderately tapered towards apex, lower tooth shorter than upper tooth. Maxillary palpus reaching to ventral part of epicnemial carina.

###### Mesosoma:

Pronotal transverse groove without median longitudinal ridge. Epomia absent. Mesoscutum smooth and shiny with very sparse and short setae (Fig. 12). Median lobe of mesoscutum without median longitudinal groove. Notauli slightly indicated anteriorly. Prescutellar groove without traces of longitudinal carinae. Scutellum moderately convex, polished and smooth or very sparsely punctate, lateral carinae strong, extending about 0.8–0.9 times length. Mesopleuron completely smooth and polished. Mesopleural impression below speculum consisting of an isolated pit some distance in front of mesopleural suture. Sternaulus weak on anterior 0.3, almost absent on hind 0.7. Epicnemial carina reaching 0.8 time height of mesopleuron, weak or absent at upper margin. Posterior transverse carina of mesosternum widely interrupted in front of each mid coxa, laterally forming low flat crest. Areolet of fore wing open. Marginal cell 2.6–2.7 times as long as deep. Ramulus absent. Vein 2*m-cu* weakly inclivous, with two bullae. Vein *cu-a* opposite *Rs+M* or slightly basal. Abscissa of *Cu_1_* between 1*m-cu* and *Cu_1a_* 2.2 times longer than *Cu_1b_*, both clearly inclivous. Hind wing with *M+Cu* moderately curved at apical 0.45. Abscissa of *M+Cu* between *M* and *Cu_1_* strongly inclivous, 1.3–1.4 times as long as *cu-a* which is strongly reclivous. Hind femur about 5.0–5.3 as long as high. Propodeum with anterior transverse carina strong and complete, posterior transverse carina absent centrally, a broad low flat crest, lateral longitudinal carina only present apically, distad of crests. Lateromedian carina partially present in area basalis. Area superomedia absent. Pleural carina rounded and strong. Submetapleural carina forming anterior strong flat crest. Juxtacoxal carina absent. Propodeal spiracle elongate.

###### Metasoma:

First metasomal tergite smooth, polished with some sparse setiferous punctures dorsally. Median dorsal and lateral carinae absent. Postpetiole about 0.7 times as long as maximum width (measured dorsally). Second and remaining tergites polished, with very weak dense setiferous punctures. Gastrocoelus wider than long, thyridium finely granulate. Ovipositor straight, with nodus and five dorsal apical teeth on upper valve, lower valve with three oblique notches and 4–5 small complete and transverse apical teeth. Ovipositor sheaths 0.9–1.0 times length of hind tibia.

###### Colour:

Body yellowish orange. Flagellum brown to blackish with a white band on flagellomeres 4–7. Sometimes mandibular teeth and hind tarsus slightly infuscated. Wing membrane with fine yellowish tinge (Fig. 7).

##### Male:

Body length 4.1–6.0 mm. Head 0.65–0.73 mm long, 1.2–1.3 mm wide. Mesosoma 1.9–2.0 mm long, 0.9–1.0 mm wide (at widest point of mesoscutum). Fore wing 4.3–4.7 mm long. Petiole 0.9–1.0 mm long.

Similar to female except as follows:

###### Head:

Transverse, 1.8–1.9 times as wide as long. Antenna with 24–26 flagellomeres. Flagellum slightly and uniformly tapered to apex. First flagellomere 4.6–5.1 times as long as maximum width. Tyloids narrow and elevated on flagellomeres 10(11)–13(14) (Figs 28, 29), with small secretory pores. Gena 0.5–0.6 times as long as eye, upper part conspicuously more rounded (Fig. 25). Malar space about 0.3–0.4 times as wide as basal width of mandible (Fig. 21). Posterior ocellus separated from eye by about 1.2–1.3 times its diameter. Space between posterior ocelli 0.45–0.55 times their diameter.

###### Mesosoma:

Mesosoma: Marginal cell 2.7–3.0 times as long as deep. Hind femur about 5.0–5.2 as long as high.

###### Metasoma:

Metasoma: Postpetiole 0.7–0.9 times as long as wide. Second and remaining tergites with dense, fine setiferous punctures.

###### Colour:

Colour: Antenna without white ring. Flagellum entirely dark brown with scape, pedicel, annellus and base of first flagellomere ventrally yellow, dorsally orange. Wing membrane with fine yellowish tinge (Fig. 6).

#### Material examined.

**Type material.** Holotypefemale with labels as follows: Argentina, Horco Molle near Tucumán, 7-13-III-1966, Lionel Stange (AEIC). Paratypes: Argentina, 1 ♀, Horco Molle near Tucumán, 1-I-1966, H. & M. Townes; 1 ♂, same locality, 8-15-I-1966, H. & M. Townes (all AEIC); 1 ♂, same locality, 15-19-I-1966, Lionel Stange (CEUA); 1 ♂, same locality, 18-I-1966, H. & M. Townes; 1 ♀, same locality, 7-13-III-1966, Lionel Stange; 1 ♂, 11 Km W. Las Cejas Tucumán, 3-18-XII-1966, Lionel Stange; 1 ♂, same locality, 7-26-III-1967, Lionel Stange; 1 ♂, same locality, 16-29-IV-1967, Lionel Stange; 1 ♂, same locality, 22-II/8-III-1968, Lionel Stange; 1 ♂, same locality, 9-III/11- IV-1968, Lionel Stange (all AEIC); 1 ♂, Jujuy, 13-I-1966, H. & M. Townes (CEUA); 1 ♂, same locality, 14-I-66, H. & M. Townes. Brazil, 3 ♀♀, M. G. Cáceres, XI-1984, M. Alvarenga; 1 ♀, 2♂♂, Caruaru, 900m, IV-1972, M. Alvarenga; 1 ♀, 1 ♂, Jatai, Goiás XI-1972, F. M. Oliveira; 1 ♂, Silva Jardin, Rio de Janeiro, VIII-1974, F. M. Oliveira (all AEIC).

#### Variation.

A male from Jujuy, Argentina (14-I-1966), has the hind half of the head, pronotum and anterior part of the mesoscutum dark brown and the mesoscutum more punctate.

#### Distribution.

Argentina, Brazil (Fig. 32).

#### Etymology.

The species name refers to the scarcely hairy, almost glabrous scutum.

### Fractipons dasyscutum sp. n.

urn:lsid:zoobank.org:act:723B9F7C-E4E3-4565-A921-9625CE31A798

#### Diagnosis.

Mesoscutum with very dense setae (Figs 10, 11). Malar space 0.6–0.8 times the width of the mandible base (Figs 16, 17). Mandible relatively concave at lower part of base, its external lower rim flatly expanded at the base, forming a translucent area (Figs 16, 17). Head in lateral view with upper part of gena straight and abruptly reduced (Figs 22, 24). Body entirely yellow-orange (Figs 8, 9), in males rarely the hind half of the head and the front part of the mesosoma dark brown. Flagellum dark brown to black, in female with a light yellow band on flagellomeres 4–8 (Fig. 8); in the male the flagellum is usually orange over 2–3 flagellomeres, never with a white band (Fig. 9).

#### Description.

##### Female:

Body length 5.0–6.2 mm. Head 0.6–0.8 mm long, 1.3–1.6 mm wide. Mesosoma 1.9–2.4 mm long, 0.8–1.1 mm wide (widest point of mesoscutum). Fore wing 4.2–5.0 mm long. Petiole 1.0–1.3 mm long. Ovipositor sheath 1.7–1.8 mm long.

###### Head:

Transverse, 1.9–2.1 times as wide as long, mostly smooth and shiny, strongly constricted behind compound eyes. Antenna with 26–28 flagellomeres, conspicuously thickened from third flagellomere, slightly thin towards apex. First flagellomere 4.6–5.4 times as long as maximum width, flagellomeres from tenth to penultimate flattened below, in this flat area with conspicuous setiferous sensillae. Gena 0.1–0.2 times as long as eye (viewed from above), upper part in lateral view straight and abruptly reduced (Fig. 22). Occiput moderately depressed centrally. Lower face finely and densely punctate with small central prominence, clypeus rather wide, weakly convex, apical margin slightly arcuate. Malar space with wide granulate groove, about 0.7–0.8 times width of mandible base (Fig. 16). Posterior ocellus separated from eye about 1.3–1.5 times its diameter. Space between posterior ocelli 0.5–0.7 times their diameter. Occipital carina reaching base of mandible, moderately elevated ventrally, sligthly angulate medially, dorsally. Mandible moderately tapered towards apex, lower tooth shorter than upper tooth, base relatively concave ventrally, external lower rim forming flat perpendicular extension, translucent at base (Fig. 16). Maxillary palpus reaching to ventral part of epicnemial carina.

###### Mesosoma:

Pronotal transverse groove without median longitudinal ridge. Epomia absent. Mesoscutum smooth and shiny with very dense setae (Fig. 10). Median lobe of mesoscutum without median longitudinal groove. Notauli impressed, reaching level of tegula. Prescutellar groove without trace of longitudinal carinae. Scutellum moderately convex, polished and smooth or very sparsely punctate, lateral carinae strong, extending about 0.8–0.9 its length. Mesopleurom completely smooth and polished. Mesopleural impression below speculum consisting of an isolated pit which is some distance in front of mesopleural suture. Sternaulus present on anterior 0.5, evanescent towards hind half. Epicnemial carina reaching 0.7–0.9 times height of mesopleurum, at upper margin weak or absent. Posterior transverse carina of mesosternon widely interrupted in front of each mid coxa, laterally elevated as flat low crest. Areolet of fore wing open. Marginal cell 3.1–3.4 times as long as deep. Ramulus absent. Vein 2*m-cu* arched, weakly inclivous, with two bullae. Vein *cu-a* opposite *Rs+M* or slightly basal. Abscissa of *Cu_1_* between 1*m-cu* and *Cu_1a_* 1.4–1.6 times length of *Cu_1b_*, both strongly inclivous. Hind wing with *M+Cu* moderately curved at apical 0.5; abscissa of *M+Cu* between *M* and *Cu_1_* strongly inclivous, 1.0–1.3 times as long as *cu-a*, which is strongly reclivous. Hind femur about 4.8–5.2 as long as high. Propodeum with anterior transverse carina strong and complete, posterior transverse carina absent centrally and forming strong, sub-triangular, flat crest joining lateral longitudinal carina, which is only present distad of crest. Lateromedian carina partially present in area basalis. Area superomedia absent. Pleural carina rounded and strong. Submetapleural carina forming anterior strong flat crest. Juxtacoxal carina absent. Propodeal spiracle elongate.

###### Metasoma:

First metasomal tergite smooth, polished with some sparse setiferous punctures dorsally, laterally. Median dorsal and lateral carinae absent. Postpetiole about 0.7–0.8 times as long as maximum width (measured dorsally). Second and remaining tergites polished, with very weak dense setiferous punctures. Gastrocoelus wider than long, thyridium finely granulate. Ovipositor straight, with nodus and five dorsal apical teeth on upper valve, lower valve with three oblique notches and 4–5 small complete, transverse apical teeth. Ovipositor sheath 0.8–1.2 times as long as hind tibia.

###### Colour:

Body entirely yellowish orange. Flagellum dark-brown to black, banded light yellow on flagellomeres 4–8, frequently orange basally. Sometimes mandibular teeth slightly infuscated. Wing membrane with fine yellowish tinge (Fig. 8).

##### Male:

Body length 5.0–6.0 mm. Head 0.6–0.8 mm long, 1.2–1.5 mm wide. Mesosoma 1.8–2.3 mm long, 0.9–1.0 mm wide (widest point of mesoscutum). Fore wing 4.7–5.6 mm long. Petiole 0.9–1.2 mm long.

Similar to female except as follows:

###### Head:

Transverse, 1.7–2.0 times as wide as long, constricted and slightly rounded behind compound eyes. Antenna with 28–30 segments. Flagellum filiform, strongly tapered from base to apex, first flagellomere 4.2–5.2 times as long as maximum width. Tyloids on flagellomeres 10–14, laminar and widely expanded at base (Figs 30, 31), with small secretory pores. Gena about 0.1–0.3 times as long as eye (in dorsal view), upper part straight and moderately reduced (Fig. 24). Malar space about 0.6–0.8 times as wide as basal width of mandible (Fig. 17). Posterior ocellus separated from eye by about 1.1–1.3 times its diameter. Space between posterior ocelli 0.5–0.9 times their diameter.

###### Mesosoma:

Marginal cell 2.6–2.8 times as long as deep. Hind femur about 4.6–5.0 as long as high.

###### Metasoma:

Postpetiole 0.7–0.9 times as long as wide. Second and remaining tergites with dense, fine setiferous punctures.

###### Colour:

Antenna without white ring. Scape, pedicel, anellus and usually first two (three) flagellomeres orange, remainder entirely dark brown. Wing membrane with fine yellowish tinge (Fig. 9).

**Figures 26–31.**
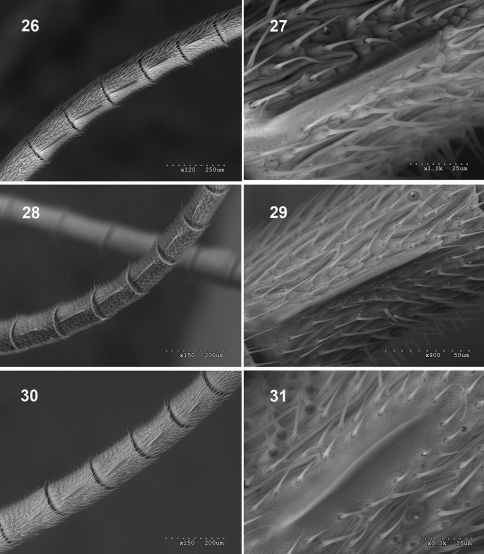
Male antenna. **26–27** Fractipons cincticornis **26** tyloids on flagellum **27** tyloid strongly magnified, **28–29** Fractipons glabriusculus sp. n.**28** tyloids on flagellum **29** tyloid strongly magnified,**30–31** Fractipons dasyscutum sp. n. **30** tyloids on flagellum **31** tyloid strongly magnified.

#### Material examined.

**Type material.** Holotype female with labels as follows**:** Costa Rica, Sector Cerro Cocori, Fca. de E. Rojas, 150 m, Provincia Limón, V-1993, E. Rojas, L.N. 286000 567500, CRI001, 347816 (INBIO). Paratypes: Costa Rica, 1 ♂, Sector Cerro Cocori, Fca. de E. Rojas, 150 m, Provincia Limón, E. Rojas, XI-1991, L.N. 286000 567500, CRI000, 466593; 2 ♀♀, same locality, E. Rojas, 28-V/15-VI-1992 L.N. 286000 567500, CRI000, 877427, CRI000, 877444; 2 ♀♀, same locality, IV-1993, E. Rojas, L. N. 286000 567500 CRI001, 346009, CRI001, 345291; 1 ♀, Est. Hitoy Cerere, 100m, R. Cerere, Provincia Limón, G. Garballo, 7-26-I-1992, L-N 184200, 648800, CRI000, 864905; 1 ♀, Estación Pitilla, 700 m, 9 Km Santa Cecilia, P. N. Guanacaste, Prov. Guanacaste, 21-III/7-IV-1993, P. Ríos, L. N. 3302000, 380200, CRI001, 387323; 1 ♂, Sector San Ramón, P. N. Guanacaste, Prov Alaju, 620 m, 27-IV/23-V-1994, E. Araya, L.N. 318100 381900, CRI001, 899301; 1 ♀, Provincia Alajuela, Upala, PN. Volcán Tenorio, Estación El Pilón, 700–800 m, 9-IX-2008, J. A. Azofeifa, Tp. amarilla, L. N. 298212 427913 #94957, INB0004171693 (all INBIO); 1 ♀, Provincia Guanacaste, Sector Murcielago, 9 Km N. del Cerro Guachipellin, 20 m, 29-VI/27-VII-1996, M Araya, Tp. Malaise, L. N. 320650 347200 #7875, CRI002, 316566; 1 ♂, Estación Biológica las Alturas, 1500 m, Coto Brus, Prov. Puntarenas, M. Ramirez, III-1992, L-S 322500, 591300, CRI000, 980023; 1 ♀, Provincia de Limón, Valle La Estrella, Banauito Lodge, 80 m, 20-21-V-2007, J. A. Azofeifa, J. Montero, Tp. Amarilla, L. N. 200889 639300, INB0004082271 (all CEUA); 2 ♂♂, S. Rosa Park, Guanacaste, 6-VI-1976, D. H. Janzen, Dry hill (1♂ CEUA, 1♂ AEIC); 1 ♂, same locality and collector, 6 -VI-1976, Riparian; 1 ♂, same locality and collector, 10-VI-1977, Riparian; 1 ♂, same locality and collector, 27-VI-1977, Riparian; 1 ♂, same locality and collector, 5-VII-1977, Riparian; 2 ♂♂, same locality and collector, 4-XI-1977, Riparian; 1 ♂, same locality and collector, 21-VI-1978, Dry hill; 1 ♂, same locality and collector, 23-VII-1978, Dry Hill (all AEIC). Panama, 1 ♂, Albrook Field Canal Zone, 25-IX-1937 (AEIC). Venezuela,1 ♂, Monagas, 27 Km SW Caripe, 300 m, 19-31-VII-1987, S. & J. Peck (CEUA). Peru**,** 1 ♂, Dept. Huanuco, Tingo María, Rio Huallaga, 9-11-VII-1974, C. Porter & L. Stange; 1 ♂, Dept. Huanuco, Cueva Las Pavas, 12-15-VII-1974, same collector (all FSCA).

#### Distribution.

**Figure 32.**
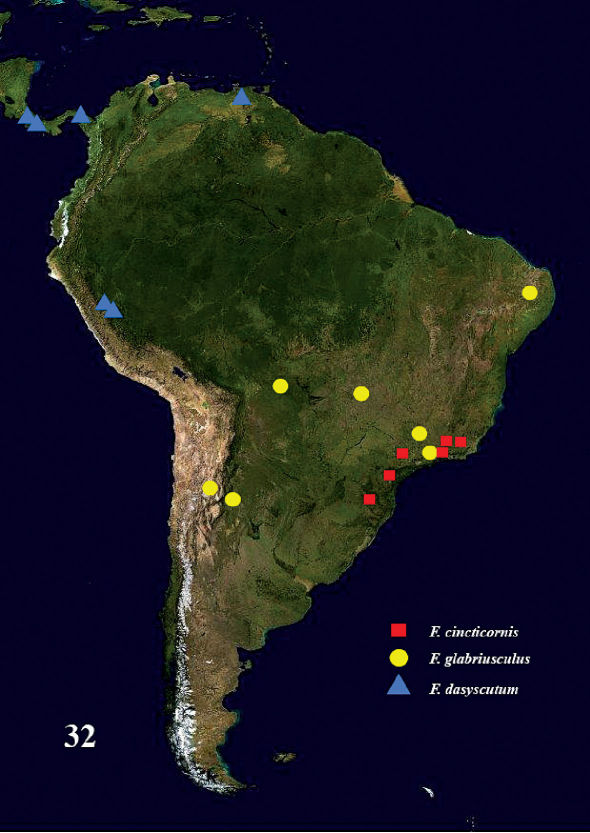
Distribution of Fractipons species.

Costa Rica, Panama, Peru, Venezuela (Fig. 32).

#### Etymology.

The species name refers to the densely hairy *scutum* (from “*dasy*” in Greek, meaning shaggy, markedly hairy).

## Supplementary Material

XML Treatment for Fractipons

XML Treatment for Fractipons cincticornis

XML Treatment for Fractipons glabriusculus

XML Treatment for Fractipons dasyscutum
